# The biochemical and growth-associated traits of basil (*Ocimum basilicum* L.) affected by silver nanoparticles and silver

**DOI:** 10.1186/s12870-024-04770-w

**Published:** 2024-02-06

**Authors:** Shahla Hashemi Shahraki, Tayebeh Ahmadi, Babak Jamali, Mehdi Rahimi

**Affiliations:** 1 Department of Biology, Faculty of Science, University of Sistan and Baluchestan, Zahedan, Iran; 2 Department of Medical Laboratory Science, College of Science, Knowledge University, Kirkuk Road, Erbil, 44001 Iraq; 3 Department of Agriculture, Minab Higher Education Center, University of Hormozgan, Bandar Abbas, Iran; 4 Department of Biotechnology, Institute of Science and High Technology and Environmental Sciences, Graduate University of Advanced Technology, Kerman, Iran

**Keywords:** Abiotic stress, Basil, H_2_O_2_, Silver nanoparticles, Total phenol

## Abstract

**Background:**

The biochemical and growth changes resulting from exposure of basil (*Ocimum basilicum* L.) seedlings to silver nanoparticles and silver were investigated. Over a two-week period, seedlings were exposed to different concentrations (0, 40, and 80 ppm) of silver nanoparticles and silver.

**Results:**

Our findings revealed that at concentrations of 40 and 80 ppm, both silver nanoparticles and silver nitrate led to decreased weight, root and shoot length, as well as chlorophyll a and b content. Conversely, these treatments triggered an increase in key biochemical properties, such as total phenols, carotenoids and anthocyanins, with silver nanoparticles showing a more pronounced effect compared to silver nitrate. Moreover, the levels of malondialdehyde (MDA) and hydrogen peroxide (H_2_O_2_) rose proportionally with treatment concentration, with the nanoparticle treatment exhibiting a more substantial increase. Silver content showed a significant upswing in both roots and leaves as treatment concentrations increased.

**Conclusions:**

Application of varying concentrations of silver nanoparticles and silver nitrate on basil plants resulted in reduced growth and lower chlorophyll content, while simultaneously boosting the production of antioxidant compounds. Notably, anthocyanin, carotenoid, and total phenol increased significantly. However, despite this increase in antioxidant activity, the plant remained unable to fully mitigate the oxidative stress induced by silver and silver nanoparticles.

## Introduction

Nanotechnology explores material manipulation within the 1–100 nanometer range, with a notable focus on silver nanoparticles that have experienced substantial growth due to silver's compelling nanoscale properties. This advancement holds pivotal importance for enhancing consumer goods and medical products [1].

Silver nanoparticles typically consist of 20–15,000 silver atoms, and in contrast to their bulk counterparts, they exhibit unique physical, chemical, and biological properties, attributed to free dangling bonds and a significant increase in surface area [2]. The reduction of material size to the nanometer range induces alterations in physical and chemical properties, impacting characteristics compared to larger scales [3]. Various industries, including electronics, biomedical sciences, pharmaceuticals, cosmetics, water purification, and catalytic systems, exploit silver nanoparticles for their distinctive properties [4]. However, a potential side effect is the release of silver nanoparticles into the environment, posing exposure risks for plants, the foundation of the ecosystem's food chain. Nanoparticles' physical characteristics, encompassing size, shape, chemical composition, surface alterations, reactivity, and concentration, profoundly influence their interactions with plants [5]. Beyond these, the impact of nanoparticles depends on factors such as treatment time, application method, nanoparticle type, plant species, and cultivar [6, 7].

In evaluating silver's effects on plant growth and biochemical content, both bulk silver and silver nanoparticles demonstrate beneficial and deleterious outcomes. Several studies highlight the positive impact of both forms on plant growth and physiological parameters [8, 9].

Yosefzaei et al. [10] reported that exposure to silver nanoparticles and silver led to increased proline and glucose contents, along with elevated activity of antioxidant enzymes like catalase and guaiacol peroxidase. Ejaz et al. [11] discovered that a concentration of 75 mg per liter of silver nanoparticles enhanced growth parameters in rice plants. In contrast, sorghum plants exposed to 1000 µM silver nanoparticles exhibited a significant increase in root length and catalase and ascorbate peroxidase activities, while a similar concentration of silver nitrate caused a decrease [12]. Hashemi et al. [13] demonstrated that applying silver nanoparticles at concentrations of 40 and 80 ppm increased flavonoid content in wheat.

Despite reports on the beneficial effects of bulk silver and silver nanoparticles on plant growth and physiological parameters, adverse impacts have also been observed on various plant species [14, 15]. For instance, *Brassica sp.* seedlings treated with silver nanoparticles and silver at different concentrations showed decreased growth, protein content, and photosynthesis, along with increased production of reactive oxygen species (ROS) [16]. Similar adverse effects were noted in rice, where a decrease in root length, fresh weight, and chlorophyll and carotenoid content occurred after silver nanoparticle application [17]. Moreover, silver nanoparticles reduced plant growth, micronutrients absorption, and protein and amino acid content in wheat [18].

J. Jiravova et al. [2] observed elevated malondialdehyde (MDA) and ROS levels in potato plants influenced by silver nanoparticles. The destructive mechanism of silver nanoparticles on plants is not fully understood, but it is suggested that the phytotoxic effects may result from the high accumulation of silver and excessive ROS production in plant tissues [2, 19]. According to Dobias and Bernier-Latmani [20], silver nanoparticles can oxidize and release silver ions, leading to ROS production during absorption and mobility of silver particles.

ROS, unstable molecules with unpaired electrons, are continuously produced during plant cell metabolism [21]. Under normal conditions, a balance between ROS production and neutralization by antioxidants, such as anthocyanins and phenols, is maintained [22, 23]. However, under stress conditions, this balance may be disrupted, resulting in oxidative stress. Excessive ROS production can damage cellular macromolecules and lead to lipid peroxidation in plants [24, 25].

Antioxidants like phenols and anthocyanins are stable molecules that donate electrons to neutralize ROS, reducing their damaging capacity. The concentration of anthocyanins in plants varies based on growth stages, environmental factors, plant species, and varieties [25, 26]. Numerous studies confirm that molecules like phenols and anthocyanins enhance antioxidant capacity, particularly in plants belonging to the Lamiaceae family [27, 28].

The basil plant (*Ocimum basilicum* L.), a member of the Lamiaceae family, includes the Purpurascens variety known for its high concentration of anthocyanins and phenols [29, 30]. Despite the use of basil plant extracts as stabilizing agents for synthesizing zinc oxide nanoparticles [31], the impact of bulk silver and silver nanoparticles on this plant remains unexplored. Hence, the objective of this study was to investigate the growth and biochemical parameters of the basil plant (*O. basilicum* var. purpurascens) treated with bulk silver and silver nanoparticles.

## Results

### Growth characteristics

**Fig. 1:**
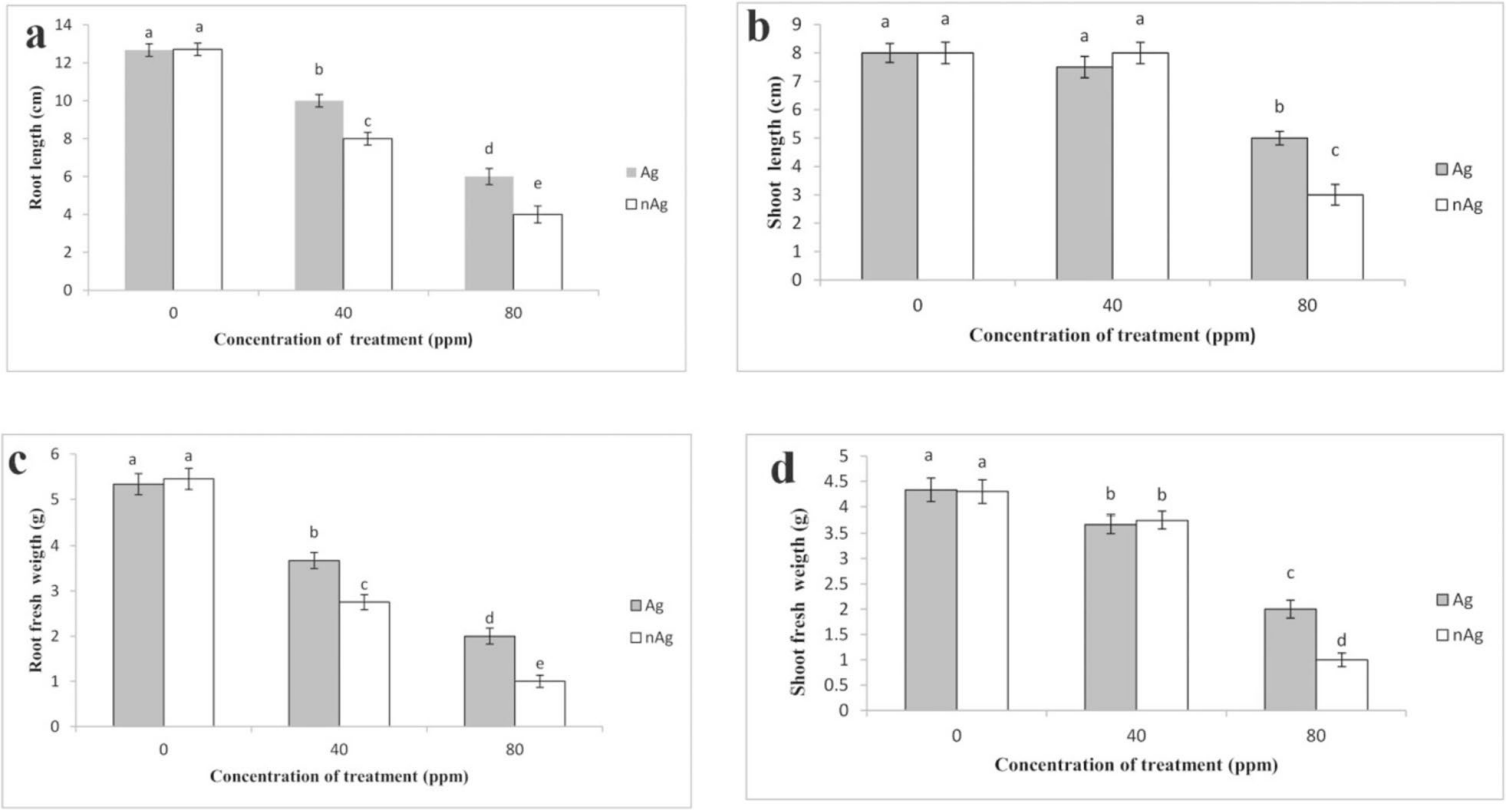
The effect of silver nitrate and silver nanoparticles on root length (**a**), shoot length (**b**), root fresh weight (**c**) and shoot fresh weight (**d**) of basil plants. Vertical bars indicate standard errors (*n* = 3). Columns with different letters represent significant differences at 5% probability

As depicted in Fig. 1, the growth-associated parameters of the basil plant, such as root and shoot length and weight, decreased with increasing concentrations of silver particles and silver nanoparticles. Silver nanoparticles at 80 ppm induced a higher reduction in growth parameters compared to silver nitrate at the same concentration. Specifically, at an 80 ppm concentration, silver particles and silver nanoparticles reduced root length by 52% and 68%, respectively, in comparison to the control group.

### Plant pigments

**Fig. 2:**
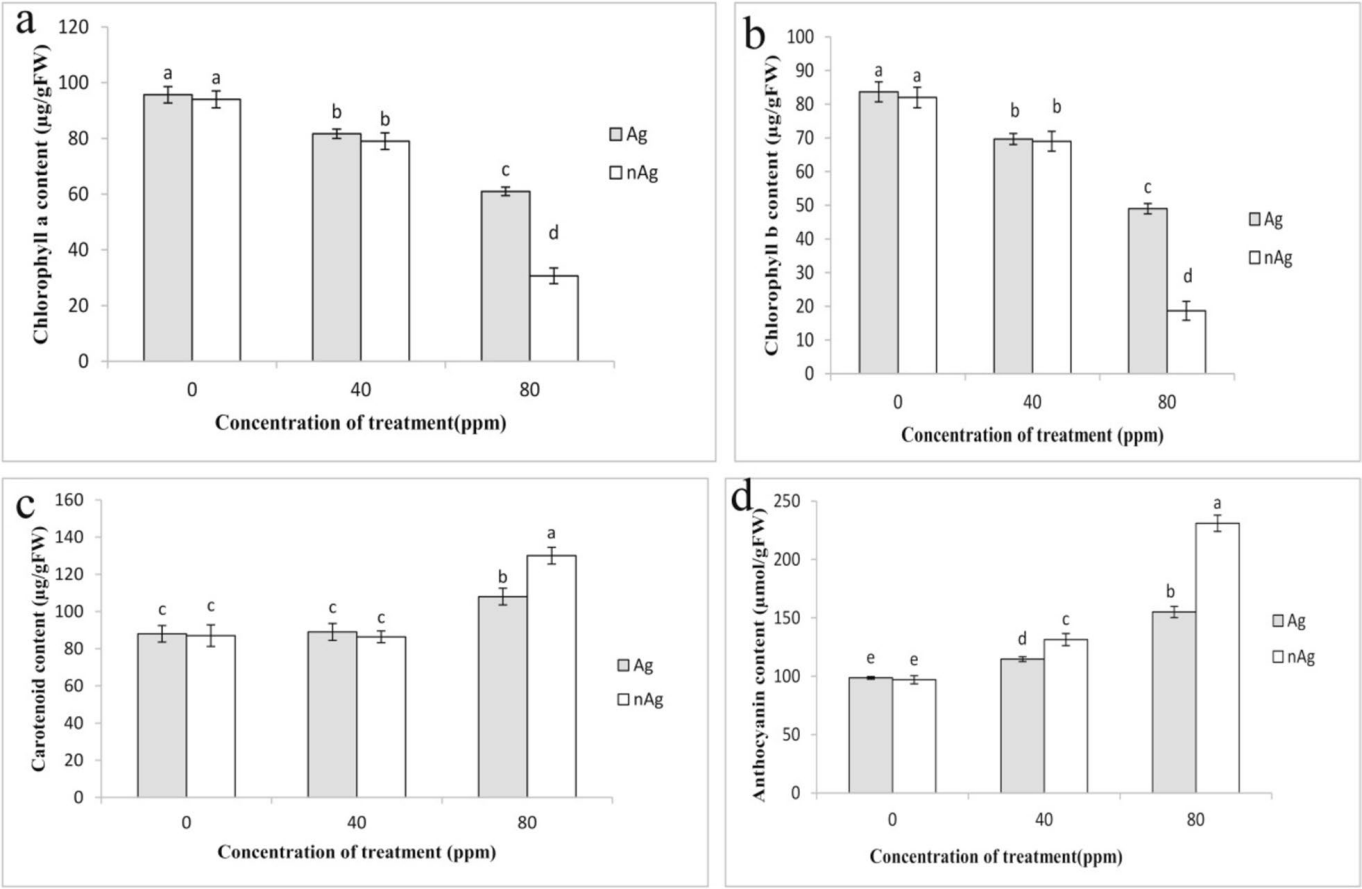
The effect of silver nitrate and silver nanoparticles on the concentration of chlorophyll a (**a**), chlorophyll b (**b**), carotenoid (**c**) and anthocyanin (**d**) in the leaves of basil palnts. Vertical bars indicate standard errors (*n* = 3). Columns with different letters represent significant differences at 5% probability

Figure 2 shows how silver atoms and silver nanoparticles affect plant pigments. As the concentration of these nanoparticles increased, the levels of chlorophylls a and b decresed, whereas the levels of anthocyanins and carotenoids increased. The concentration of chlorophyll a and b in silver particles and silver nanoparticles did not differ at a concentration of 40 ppm. However, compared to silver particles, silver nanoparticles reduced these two pigments by a higher amount at an 80 ppm concentration. The silver particles and silver nanoparticles, at a concentration of 80 ppm, resulted in a 36% and 67% decrease in chlorophyll a, respectively, compared to control plants. Chlorophyll b decreased by 77% at a concentration of 80 ppm silver nanoparticles, compared to control plants.

### Total phenols, MDA, and hydrogen peroxide (H_2_O_2_)

**Fig. 3:**
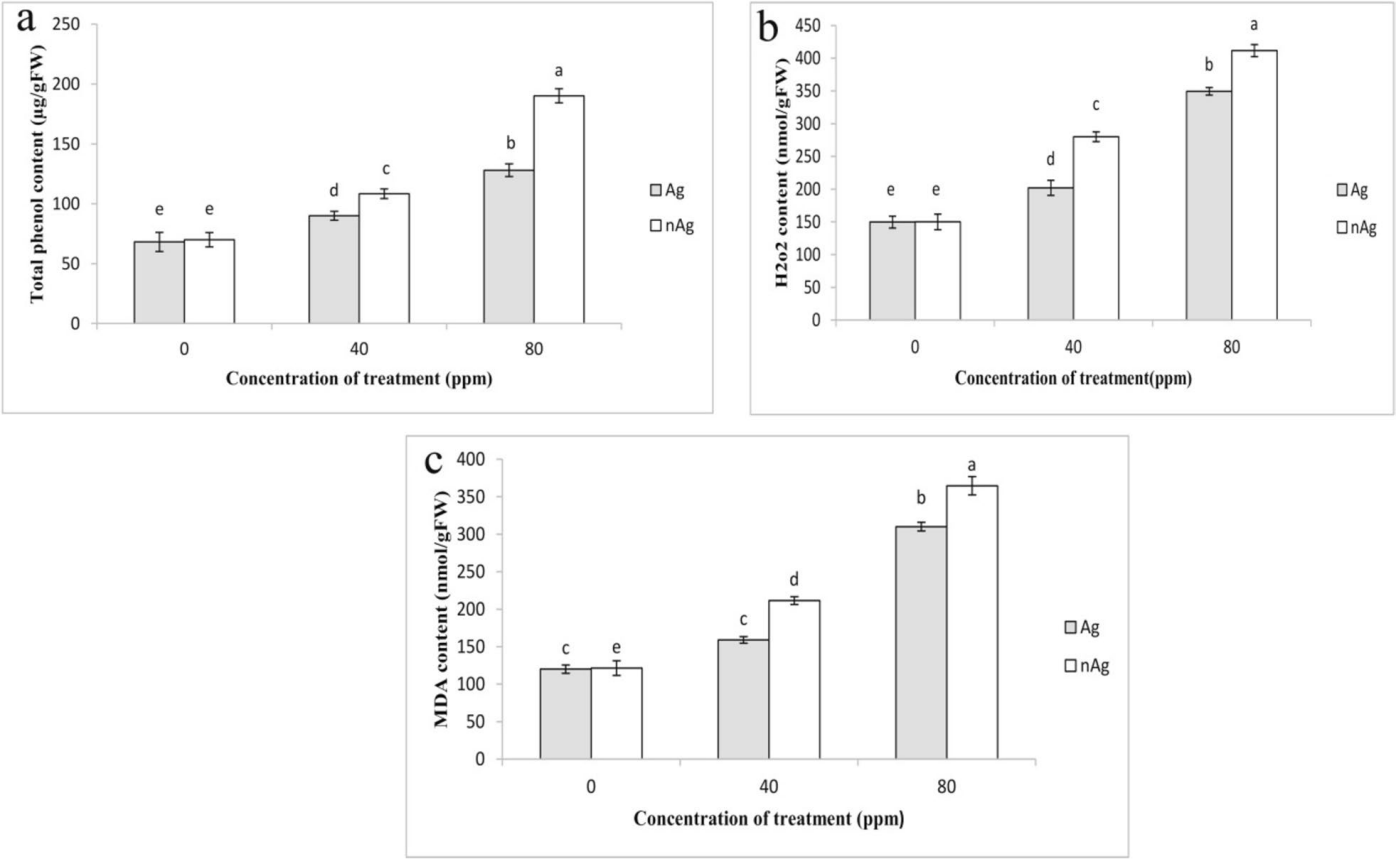
The effect of silver nitrate and silver nanoparticles on the concentration of total phenols (**a**), H2O2 (**b**) and MDA (**c**) in the leaves of basil plants. Vertical bars indicate standard errors (*n* = 3). Columns with different letters represent significant differences at 5% probability

Figure 3 shows that an increase in the concentration of silver particles and silver nanoparticles led to an increase in biochemical parameters such as total phenols, MDA, and H_2_O_2_. In particular, silver nanoparticles caused a greater increase in these parameters compared to silver nitrate, especially at a concentration of 80 ppm. At a concentration of 80 ppm of silver nanoparticles, total phenols, MDA, and H_2_O_2_ increased by 17%, 20%, and 17%, respectively, compared to the control.

### Silver content

**Fig. 4:**
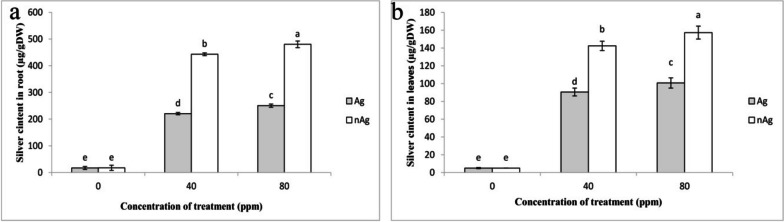
The impact of silver nitrate and silver nanoparticles on silver concentration in basil plants' roots (**a**) and leaves (**b**). Vertical bars indicate standard errors (*n* = 3). Columns with different letters indicate significant differences at a 5% probability level

As the treatment concentration increased, there was a rise in silver content within both the roots and leaves (Fig. 4). The silver content in plants treated with silver nanoparticles exceeded that in plants treated with silver nitrate. Applying 40 ppm and 80 ppm of silver nanoparticles resulted in silver content increases of 23% and 35%, respectively, when compared to the control.

## Discussion

Nanoparticles, defined as particles smaller than 100 nm, exhibit distinctive physical and chemical properties owing to their reduced size, unique structure, and surface characteristics, setting them apart from their bulk counterparts [32, 33]. Among the different types of nanoparticles, silver nanoparticles stand out as important elements used in various industries, particularly in medicine, due to their unique properties. However, the widespread utilization of silver nanoparticles results in their release into the ecosystem. This dissemination has consequential effects on plants, which play a vital role in the ecosystem [34].

The impact of silver nanoparticles on plants manifests in intracellular changes, influenced by the plant's sensitivity or tolerance to these nanoparticles [35]. Plant responses to nanoparticle exposure encompass a spectrum of morphological and physiological alterations. Key factors influencing the extent of these changes include the chemical composition, size, concentration, surface coating, reactivity of the nanoparticles, treatment duration, application method, and the type of plant species or cultivar [7, 36, 37]. Growth parameters and content of photosynthetic pigments provide insight into how plants react to stress caused by nanoparticles [38, 39]. Al-Huqail et al. [40] found that silver nanoparticles have the potential to inhibit chlorophyll production in leaves, consequently impacting a plant's photosynthetic capabilities. This aligns with the research conducted by Vishwakarma et al. [41], who observed a reduction in photosynthetic pigments in mustard plants due to the generation of ROS induced by silver nanoparticle treatment.

Growth and chlorophyll content have been observed to be negatively impacted by the presence of silver and silver nanoparticles in our experiment. The reduction was found to be more significant in the presence of silver nanoparticles compared to silver alone. The observed reduction in growth parameters appears to be linked to a decline in chlorophyll content induced by the treatment with silver and silver nanoparticles. The generation of H_2_O_2_, an example of ROS, coupled with an elevated absorption of silver ions, seems to contribute to the diminished chlorophyll levels in plants exposed to silver and silver nanoparticles. Consequently, the detrimental effects of silver nanoparticles, in comparison to silver, on both growth and chlorophyll content are more pronounced, likely attributed to the heightened absorption of silver ions and the increased production of ROS.

Furthermore, silver ions can impact photosynthesis by competitively displacing copper in plastocyanin. Plastocyanin is a soluble protein found in the lumen of chloroplast thylakoids that binds to copper. Electrons are transferred from cytochrome b6/f to PSI through plastocyanin as an electron carrier [42, 43].

Jansson and Hansson (2008) reported that silver ions have the capacity to inhibit the electron transfer process from plastocyanin to PSI [44].

When silver ions bind to plastocyanin and compete with copper for binding sites, they disrupt or inactivate the photosynthetic electron transport process, leading to the production of ROS [45]. Plant cells overproduce ROS due to the activation of silver nanoparticle and silver phytotoxicity, which causes oxidative stress. Multiple studies have shown that the formation of four distinct ROS in plants, including singlet oxygen, superoxide, H_2_O_2_, and hydroxyl radicals, is significantly increased when plants are exposed to silver nanoparticles and silver [46].

In organelles including chloroplasts, mitochondria, and peroxisomes, ROS is created naturally as a byproduct of normal metabolic processes [47]. However, under stressful conditions, an excessive amount of ROS is produced, and this ROS, through electron transfer, significantly oxidises plant biomolecules and cellular structures. Arruda et al. [48] reported that excessive ROS generation induced by exposure to silver nanoparticles might result in oxidative stress, which can induce cell death and impair plant growth. Oxidative stress may result in lipid peroxidation, protein damage, DNA damage, and permeability damage to cell membranes.

MDA is a significant byproduct of lipid peroxidation and serves as an indicator of the extent of lipid peroxidation in suboptimal growth conditions [49]. Our research revealed a direct correlation between the concentrations of silver and silver nanoparticles and the content of H_2_O_2_ and MDA. As the concentration of silver and silver nanoparticles increased, there was a corresponding rise in the levels of H_2_O_2_ and MDA. Notably, the increase was more pronounced in the case of silver nanoparticle treatment.

Silver nanoparticle treatment at 500 and 2000 ppm boosted the development of MDA in soybeans, as observed by De La Torre-Roche et al. [50]. Similar to this, Nair and Chung [51] found that exposing Arabidopsis plants to silver nanoparticle concentrations of 0.2, 0.5, and 1 ppm significantly increased lipid peroxidation. Further research revealed that exposure to silver nanoparticle concentrations of 0.5 and 1 ppm significantly increased the production of H_2_O_2_ and lipid peroxidation in rice shoots and roots [17].

Plants possess inherent antioxidant defense systems designed to counteract the detrimental effects of ROS. These defense mechanisms encompass both non-enzymatic antioxidants, such as thiols, ascorbate, glutathione, phenols, and anthocyanins, as well as enzymatic antioxidants, including ascorbate peroxidase, catalase, and superoxide dismutase. Together, these components work synergistically to neutralize and regulate ROS levels, safeguarding the plant from oxidative stress and maintaining cellular integrity [52].

Anthocyanins and phenols frequently act as a non-enzymatic antioxidant to combat ROS and chelate metals in stressful situations [53].

In our study, the plants treated with both silver and silver nanoparticles showed increased concentrations of anthocyanins, carotenoids, and total phenols compared to the control group. The increase in these antioxidant compounds was particularly pronounced in the treatment with silver nanoparticles, underscoring its greater impact on the accumulation of anthocyanins, carotenoids, and total phenols in the plants.

According to a study by Syu et al. [54], treating Arabidopsis seedlings with silver nanoparticles significantly boosted anthocyanin and phenols accumulation. Similar outcomes in terms of anthocyanin accumulation were seen in turnip after exposure to higher quantities of silver nanoparticles [55].

Additionally, anthocyanins, phenols, and other non-enzymatic antioxidants like carotenoids participate in plant antioxidant defence responses to silver nanoparticles. Carotenoids have the ability to stimulate antioxidant activity and may lessen the harmful effects of ROS. After being exposed to silver nanoparticles, rice showed a large increase in the level of carotenoids, showing that plants utilize carotenoids to mitigate the ROS effects induced by silver nanoparticles [56].

In this study, despite the presence of anthocyanin, phenol, and carotenoid antioxidants, the effective removal of ROS generated under the stress of silver and silver nanoparticles was not sufficient. Consequently, ROS induced damage to fatty acids, leading to the production of MDA, with higher MDA content observed in the silver nanoparticle treatment compared to silver.

Supporting these findings, Okaroum et al. (2013) proposed that the release and accumulation of silver from silver nanoparticles elevate ROS production in *Lemna gibba* [57]. In our study, the accumulation of silver in both roots and leaves of plants exposed to silver and silver nanoparticles increased, with a more significant rise observed in the silver nanoparticle treatment. Consistent with our results, Arabidopsis plants exposed to silver nanoparticle treatment also demonstrated a higher accumulation of silver compared to those exposed to silver alone [58]. These outcomes highlight the potential implications of silver nanoparticles in triggering ROS-related stress and silver accumulation in plants.

## Conclusions

Our research investigated the impacts of silver nitrate and silver nanoparticles on basil* (Ocimum basilicum* var. *Purpurascens*) plants. The results revealed a significant decrease in growth-related traits such as root and shoot length, as well as fresh weight, following these treatments.

The decrease in growth parameters observed in both silver and silver nanoparticle treatments suggests a potential impact of oxidative stress on the plants. Specifically, the silver nanoparticle treatment triggered a stronger oxidative stress response than silver alone, as evidenced by the higher levels of H2O2 and MDA. This heightened oxidative stress in the plants treated with silver nanoparticles can be attributed to increased silver absorption and elevated ROS production.

Although non-enzymatic antioxidant responses were stimulated in the plants treated with silver and silver nanoparticles, the antioxidant activity proved to be insufficient to completely neutralize ROS. These results emphasize the potentially detrimental effects of silver particles, particularly nanoparticles, on the growth and physiological parameters of basil plants. In light of these findings, the authors recommend implementing measures to regulate the release and accumulation of silver and silver nanoparticles in the environment and emphasize the importance of environmental management in mitigating potential adverse effects on plant health.

## Materials and methods

### Plant materials and treatments

This study complied with relevant institutional, national, and international guidelines and legislation of Iran. The seeds of plant material were obtained from Pakan Seed Company of Isfahan. No special permissions were necessary to collect samples. Otherwise, the plant materials used and collected in the study comply with Iran’s guidelines and legislation. The plant studied in this research was the purple basil plant and the variety used was Purpurescens.

**Fig. 5:**
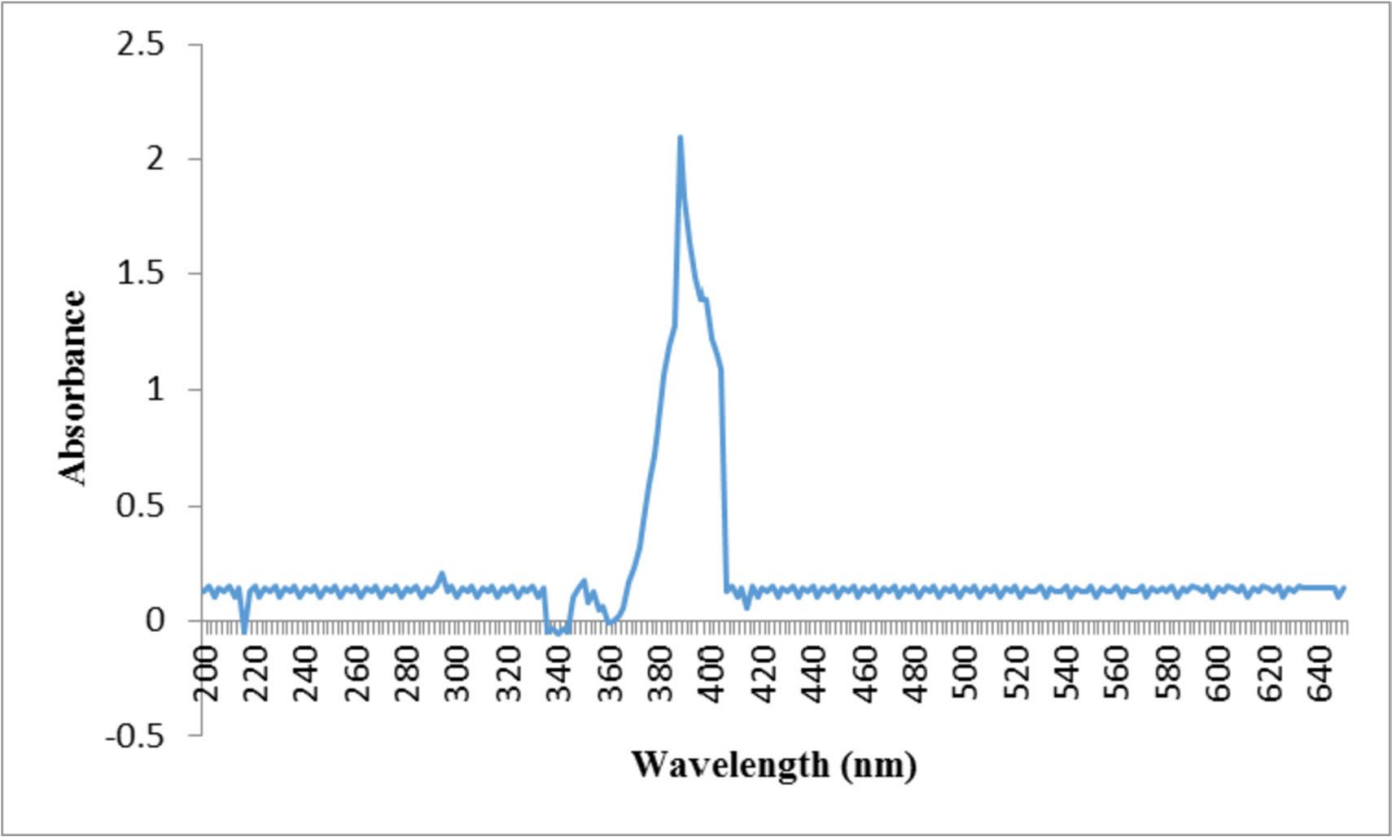
UV–vis spectra of silver nanoparticles

**Fig. 6:**
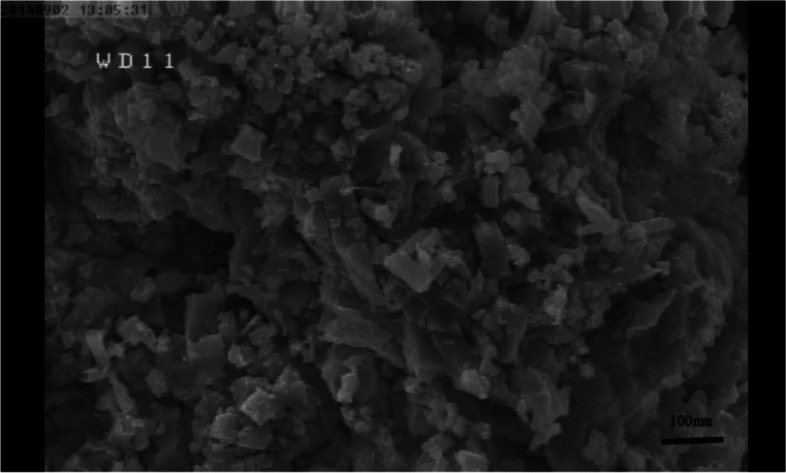
SEM micrograph of the silver nanoparticles

The basil seeds were disinfected for six minutes with a 0.5% sodium hypochlorite solution. After being rinsed twice with distilled water, the seeds were sown in pots filled with perlite. They were then incubated in a greenhouse under controlled conditions, including a temperature of 25 ± 2 °C, a 16/8 light/dark cycle, a relative humidity of 60%, and a light intensity of 120 µmolm^−2^s^−1^light intensity. The seedlings were irrigated with Hoagland's solution for two weeks following germination. Then, therapies utilising silver nitrate (representative of Bulk Silver) and silver nanoparticles were used. According to the manufacturer's data, the average size of silver nanoparticles was 20 nm, and the nanoparticles were homogeneous. UV spectral analysis (using a Scan Drop-type product from Analytik Jena, Germany) and scanning electron microscopy (Model JSM 6390LV, JOEL, USA) of the nanoparticles confirmed the manufacturer's information. UV spectroscopy showed the absorption maximum at a wavelength of 383 nm, and the SEM image revealed that the nanoparticles were homogeneous (Figs. 5 and 6). This experiment was done based on completely randomized design with three replication.

The Hoagland's nutritional solution was supplemented with solutions containing silver nitrate and ultrasonically sonicated silver nanoparticles (100W, 40 kHz, 30 min) at concentrations of 0, 40, and 80 ppm. After two weeks of treatment, the plants were selected for measuring growth and physiological characteristics.

### Measurement of chlorophyll, carotenoid content and growth-associated traits

After being initially cleaned with distilled water, the samples had any extra moisture absorbed by filter paper. After the roots were cut off from the soil's surface, the weight of the plant's shoot and root was measured in gram. A ruler was also used to measure the root' and shoot' length in centimetre. Measurement of chlorophyll and carotenoid content in leaves was performed using the Arnon method [59] for chlorophyll and the Lichtenthaler method [60] for carotenoids. First, one gram of fresh leaf tissue was weighed and ground using a mortar and pestle. The resulting extract was then adjusted to a volume of 10 ml using 80% acetone. Subsequently, the extract was centrifuged for three minutes at 4 degrees Celsius and 5000 g. The absorbance of the mixture was measured using a spectrophotometer at wavelengths of 645, 663, and 470 nm after transferring a portion of the extract into a cuvette. Finally, the concentrations of chlorophylls a and b, total chlorophyll, and carotenoids were determined in micrograms per gram of plant tissue weight using the following formulas:

1 $$\mathrm{Chlorophyll}\;\mathrm a\;\left(\text{mg}/\text{gfw}\right)=\left(\left(12.7\times\text{D}663\right)-\left(2.69\times\text{D}645\right)\right)\times\text{V}/1000\times\text{W}$$

2 $$\mathrm{Chlorophyll}\;\mathrm b\;\left(\text{mg}/\text{gfw}\right)=\left(\left(22.9\times\text{D}645\right)-\left(4.93\times\text{D}663\right)\right)\times\text{V}/1000\times\text{W}$$

3 $$\mathrm{Total}\;\mathrm{chlorophyll}\;\left(\text{mg}/\text{gfw}\right)=\left(\left(20.2\times\text{D}645\right)+\left(8.02\times\text{D}663\right)\right)\times\text{V}/1000\times\text{W}$$

4 $$\mathrm{Carotenoids }\left({\text{mg}}/{\text{gfw}}\right) = \left(1000 \times {\text{D}}470 -1.82 \times \mathrm{chla }-85.02 \times {\text{chlb}}\right)/198 \times {\text{V}}/1000 \times {\text{W}}$$

### Total phenolic compounds in leaves

The Sonald and Laima technique [61] was employed to determine the total phenolic content of the leaves. One gram of the sample was mixed with 5 ml of 95% ethanol and left in the dark for 24 h. Afterward, 1 ml of the extract was combined with 1 ml of 1N HCl and 1 ml of 5% sodium carbonate, then diluted with distilled water to reach a final volume of 5 ml. Following an hour in the dark, the absorbance of each sample was measured at a wavelength of 725 nm. The concentration of total phenolic compounds was then calculated in mg per gram of plant tissue weight using a standard curve.

### H_2_O_2_ content

The H_2_O_2_ level was determined using the Velikova et al. [62] technique. Plant leaves were homogenised, then rinsed with 1.0% trichloroacetic acid. The extract was centrifuged at 10,000 g for 15 min at 4°C using a refrigerated centrifuge (Centrifuge 5804R, Germany). Then, 5.0 ml of supernatant and 5.0 ml of 10 mM potassium phosphate buffer (pH 7.0) were combined with 1.0 ml of 1 M potassium iodide solution. The absorbance was measured at a wavelength of 390 nm. The concentration of H_2_O_2_ in each sample was calculated and reported as micromoles per gram of plant tissue weight using the extinction coefficient of M^−1^cm^−1^ 0.28.

### MDA content

Measurement of Malondialdehyde (MDA) in whole leaf was performed using the Heath and Packer [63] method.

### Anthocyanin content

The concentration of anthocyanins was determined using the Wagner method [64]. In a mortar, 1.0 g of the material was completely crushed with 10 ml of acidic methanol (pure hydrochloric acid and methanol in a volumetric ratio of 99:1). The extract was then put into test tubes with screw-on caps and left for 24 h at 25°C in complete darkness. The extract was centrifuged at 4000 g for 10 min, and the supernatant's absorbance at 550 nm was measured. Using an extinction coefficient of 33000 M^−1^ cm^−1^, the concentration was estimated, and the results were given in micromoles per gram dry weight.

### Silver content

To measure the silver content, the roots and leaves of control and treated plants (exposed to silver nitrate and silver nanoparticles) were collected and completely washed to remove silver nanoparticles attached to the root surface. The samples were then dried in an oven at 70°C for 72 h and digested in nitric acid in a microwave oven. After digestion, the samples were diluted and analyzed using inductively coupled plasma mass spectrometry (ICP-MS, Agilent 7500a, USA).

### Statistical analysis

The experiment was conducted based on completely randomized design, with three replications. In each replication, measurements were made on five random samples and their average was considered as the average of that replicate. The mean values shown in the figures represent the average of these three independent biological replicates, with the associated standard error. The data were analysed using the ANOVA test and SPSS software. The Duncan's test was used to compare means at a 5% level of probability.

## Data Availability

The data of this study are available from the corresponding author upon request.
